# Structure cristalline et analyses thermique et de surface Hirshfeld du diperchlorate de 4-aza­niumyl-2,2,6,6-tétraméthylpipéridin-1-ium

**DOI:** 10.1107/S2056989017012695

**Published:** 2017-09-12

**Authors:** Hammouda Chebbi, Abdessalem Boumakhla, Mohamed Faouzi Zid, Abderrahmen Guesmi

**Affiliations:** a Université de Tunis El Manar, Faculté des Sciences de Tunis, Laboratoire de Matériaux, Cristallochimie et Thermodynamique Appliquée, 2092 El Manar II, Tunis, Tunisia; b Institut Préparatoire aux, Etudes d’Ingénieurs de Tunis, rue Jawaher Lel Nehru, 1089 Montfleury, Tunis, Tunisia

**Keywords:** perchlorate, organic-inorganic hybrid, crystal structure, graph-set motifs

## Abstract

The synthesis of 4-aza­niumyl-2,2,6,6-tetra­methyl­piperidin-1-ium diperchlorate, C_9_H_22_N_2_^2+^·2ClO_4_^−^, was carried out from an aqueous reaction of perchloric acid with 4-amino-2,2,6,6-tetra­methyl­piperidine. This compound was characterized by TGA–DSC analysis and single-crystal X-ray diffraction. The piperidine ring of the dication adopts a chair conformation and the orientation of the C—NH_3_ bond is equatorial. One of the two crystallographically independent perchlorate anions exhibits disorder [occupancies 0.625 (7) and 0.375 (7)]. The crystal packing is constituted by a succession of mixed layers parallel to the (-102) plane, made up of C_9_H_22_N_2_^2+^ dications and ClO_4_^−^ anions. These ions are linked by normal and bifurcated N—H⋯O hydrogen bonds with *R*_4_^4^(12) graph-set motifs, generating a two-dimensional network. The inter­molecular inter­actions in the crystal structure were qu­anti­fied and analysed using Hirshfeld surface analysis.

## Abstract 2

A new organic perchlorate (C_9_H_22_N_2_)[ClO_4_]_2_ was synthesized by slow evaporation at room temperature and its crystal structure was determined. This compound was characterized by TGA–DSC and Hirshfeld surface analysis.

## Contexte chimique   

Les matériaux hybrides ‘organique–inorganique’ sont l’objet d’un intérêt sans cesse croissant permettant d’allier à la fois certaines propriétés d’un matériau inorganique (ou d’une molécule inorganique), et certaines propriétés d’un polymère (ou d’une molécule organique). Cette symbiose entre deux mondes de la chimie trop longtemps considérés comme antagonistes peut aussi amener à des propriétés complètement nouvelles, et ouvre un vaste champ d’investigations pour le chimiste.
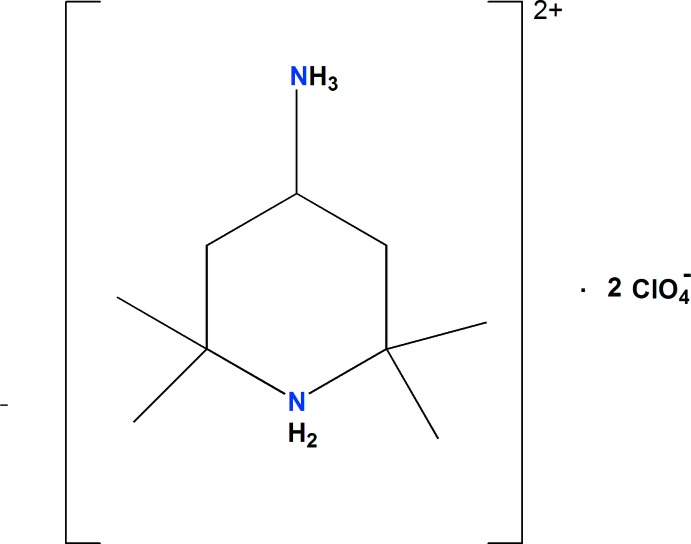


Les applications de ces matériaux hybrides couvrent des champs variés telle que les propriétés de résistance mécanique (Mammeri *et al.*, 2005 ▸) et le doublage de fréquence (Mackenzie, 1993 ▸) d’où leurs applications dans le domaine de l’électronique. De leur côté, les composés hybrides à base de perchlorates ont été particulièrement étudiés du fait des propriétés physiques intéressantes qu’ils présentent comme (1) la ferroélectricité: perchlorate de pyridinium (Czamecki *et al.*, 1994 ▸), perchlorate de pyridin-4-ylméthanaminium (Cui *et al.*, 2016 ▸), perchlorate de guanidinium (Drozd & Dudzic, 2013 ▸) et (2) l’optique non-linéaire: perchlorate d’anilinium (Vivek *et al.*, 2015 ▸), perchlorate de *p*-nitro­anilinium (Bouchouit *et al.*, 2008 ▸), perchlorate de l-leucinium (Marchewka & Drozd, 2013 ▸). La condition fondamentale pour le test de l’optique non-linéaire est que le composé soit non centrosymétrique.

Dans la chimie des perchlorates et en particulier les perchlorates à cations organiques, plusieurs structures décrites dans la littérature (Dai, 2008 ▸; Zhang, 2009 ▸; Anitha *et al.*, 2013 ▸; Direm *et al.*, 2015 ▸) présentent une cristallochimie particulière avec des liaisons hydrogène assurant leurs cohésions.

Ce travail porte sur un perchlorate à cation organique qui résulte de l’inter­action entre l’acide perchlorique et une molécule organique possédant deux doublets électroniques libres: 4-amino-2,2,6,6-tétraméthylpipéridine. Il s’agit du composé (C_9_H_22_N_2_)[ClO_4_]_2_ (I). Nous présentons ici sa structure cristalline et ses caractérsitiques thermique (TG–DSC) ainsi que l’analyse de sa surface Hirshfeld.

## Analyse structurale   

**Figure 1:**
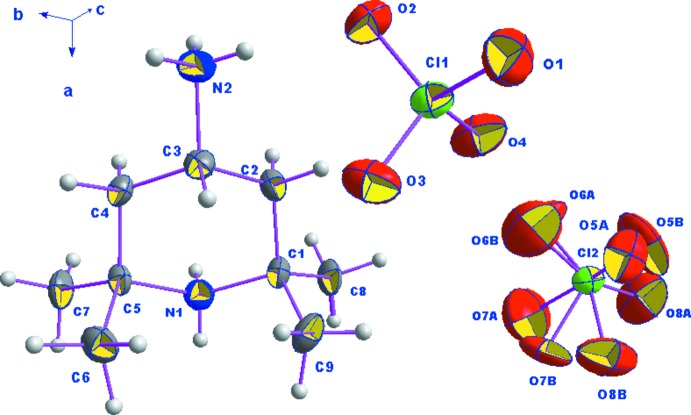
L’unité asymétrique de (I). Les ellipsoïdes d’agitation thermique ont 30% de probabilité d’existence.

**Figure 2:**
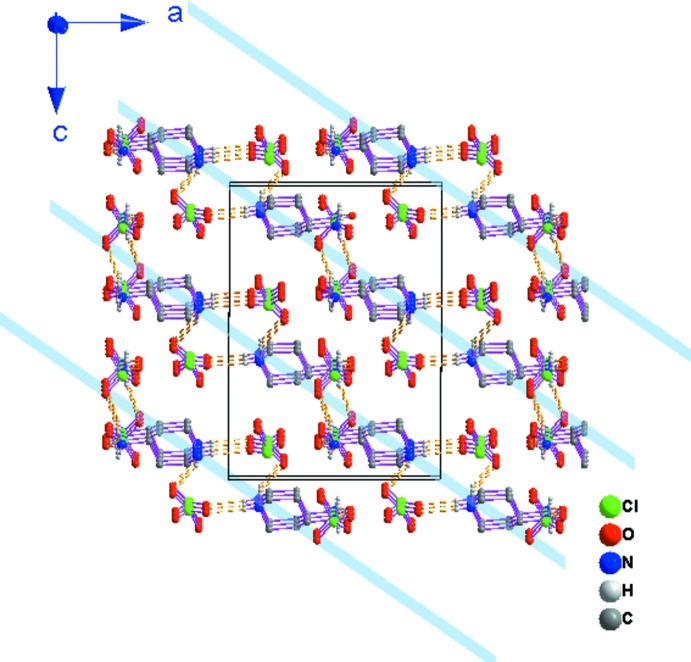
Vue en perspective de la structure bidimensionnelle du composé (I) montrant les couches mixtes parallèles au plan (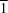
02). Pour la clarté de la figure on a representé uniquement les atomes d’oxygène possédant le taux d’occupation le plus élevé dans le perchlorate désordonné et les atomes d’hydrogène des groupements –NH_2_^+^ et –NH_3_^+^.

La détermination structurale du composé diperchlorate de 4-aza­niumyl-2,2,6,6-tétraméthylpipéridin-1-ium de formule (C_9_H_22_N_2_)[ClO_4_]_2_ (I), a permis d’établir le modèle structural, dont l’unité asymétrique est constituée d’un cation organique diprotoné et de deux anions perchlorate dont un est désor­donné (Fig. 1 ▸). La jonction entre ces entités est assurée par des liaisons hydrogène modérées de type N—H⋯O (Jeffrey, 1997 ▸). La structure cristalline de (I) est constituée par une succession de couches mixtes formées d’anions et de cations, parallèles aux plans (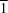
02) (Fig. 2 ▸).

Dans le composé étudié un des anions, Cl2O_4_^−^, présente un désordre qui se manifeste par l’agitation thermique de tous les atomes d’oxygène. Ainsi, chaque atome d’oxygène se trouve partagé entre deux sites cristallographiques avec des taux d’occupation de 0,625 (7) et 0,375 (7). Une recherche bibliographique sur les composés perchloratés a révélée l’existence de quatre composés similaires présentant le même phénomène de désordre (Butcher *et al.*, 2002 ▸; Sutha Siluvai *et al.*, 2005 ▸; Kaabi *et al.*, 2010 ▸, 2011 ▸). L’environnement de l’autre atome de chlore Cl1 est tétraédrique avec une déformation considérable. En effet, le tétraèdre Cl1O_4_ contient deux liaisons Cl—O courtes qui valent respectivement 1,413 (3) et 1,422 (3) Å et deux autres plus longues 1,433 (3) et 1,438 (3) Å. Les distances les plus longues concernent les deux atomes d’oxy­gène O2 et O3 engagés dans les liaisons hydrogène. Ces valeurs sont comparables à celles du même anion étudié avec d’autres types de cations (Bendjeddou *et al.*, 2003 ▸; Avecilla *et al.*, 2005 ▸; Zhao, 2012 ▸; Gholizadeh *et al.*, 2014 ▸).

Les cations organiques (C_9_H_22_N_2_)^2+^ occupent des positions générales et assurent l’équilibre des charges négatives excédentaires portées par les anions ClO_4_^−^ et contribuent à la cohésion structurale. Le cycle pipéridine de ce cation adopte la conformation chaise avec orientation équatoriale pour la liaison C—NH_3_^+^. Dans le groupement organique, les valeurs des longueurs de liaison C—C varient de 1,522 (5) à 1,539 (5) Å; la valeur moyenne des distances C—N est égale à 1,525 (4) Å et les angles C—C—C, C—C—N, C—N—C sont compris entre 105,5 (3) et 120,7 (2)°. Ces valeurs ne présentent pas d’anomalies, elles sont comparables aux structures contenant le même cation 4-amino-2,2,6,6-tétra­méthyl­pipéridine (Huang & Deng, 2007 ▸; Mrad *et al.*, 2009 ▸; Chebbi & Driss, 2001 ▸; Chebbi *et al.*, 2014 ▸).

## Caractéristiques supra­moléculaires   

La cohésion de l’édifice cristallin est assurée principalement par sept liaisons hydrogène modérées de type N—H⋯O entre les cations et les anions (Jeffrey, 1997 ▸). Les distances et les angles décrivant les liaisons hydrogène sont donnés dans le Tableau 1 ▸.

**Figure 3:**
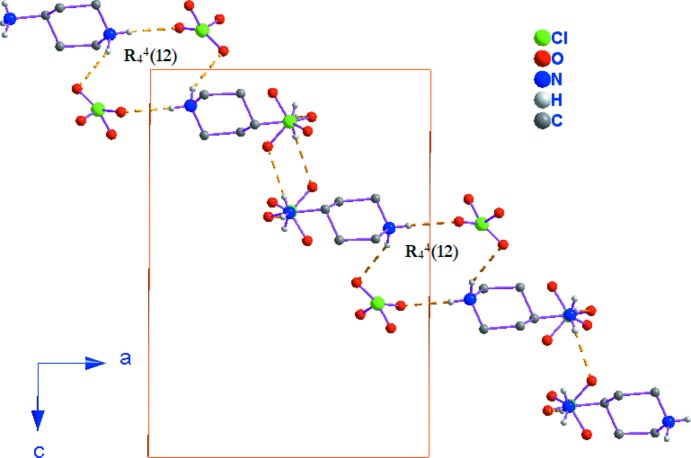
Chaîne ondulée se developant selon la direction [201] donnant lieu aux motifs de liaisons hydrogène 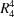
(12).

**Figure 4:**
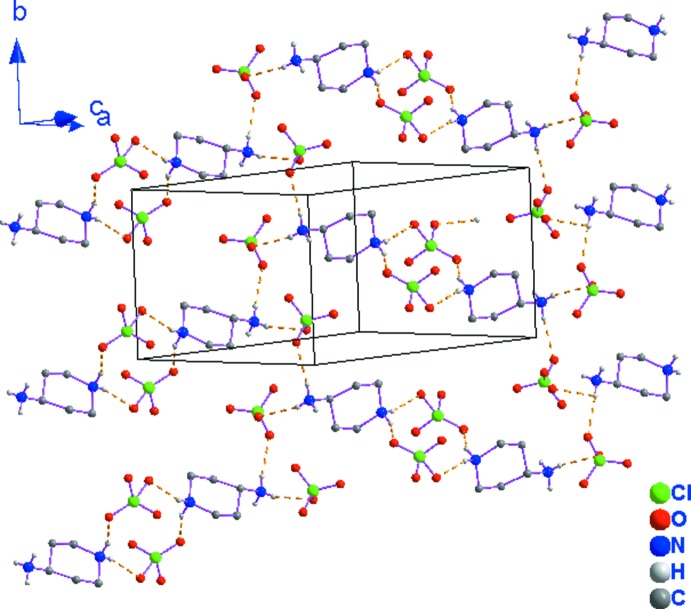
Chaînes ondulées formant une couche mixte dans la structure de (I) montrant les liaisons hydrogène de type N—H⋯O, intra- et inter-chaînes.

Les anions ClO_4_^−^ jouent un rôle important dans la cohésion de la structure du fait qu’ils participent dans les liaisons hydrogène de type N—H⋯O en acceptant des atomes d’hydrogène de la partie organique. En effet, les tétraèdres Cl1O_4_ se connectent avec les groupements –N2H_3_^+^ du dication organique pour former des chaînes ondulées se developant selon la direction [201] donnant lieu aux motifs de liaisons hydrogène 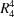
(12) (Fig. 3 ▸). Dans une couche mixte les cations (C_9_H_22_N_2_)^2+^ orientent leurs groupements –NH_2_^+^ et –NH_3_^+^ vers les atomes d’oxygène des anions perchlorate permettant ainsi d’établir des liaisons hydrogène de type N—H⋯O, intra-et inter-chaînes, qui contribuent à la cohésion bidimensionnelle du réseau (Fig. 4 ▸).

## Analyse thermique   

L’étude thermique est réalisée à l’aide d’un thermoanalyseur de type Setaram–Labsys Evo TG–DSC dans lequel un creuset vide est utilisé comme référence. L’analyse thermogravimétrique est effectuée avec 5,9 mg de produit placé dans un creuset en platine. Le chauffage se fait de l’ambiante jusqu’à 723 K avec une vitesse de 5 K min^−1^ sous atmosphère d’argon.

**Figure 5:**
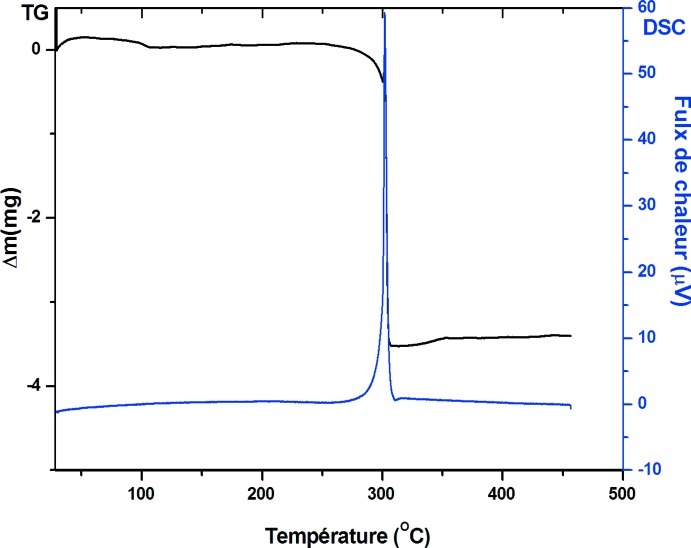
Courbes TG–DSC du composé (I).

Le thermogramme TG–DSC du composé (C_9_H_22_N_2_)[ClO_4_]_2_ est représenté sur la Fig. 5 ▸. La courbe TG révèle une seule perte de masse importante dans le domaine 543–578 K, ce qui prouve que le composé étudié est dépourvu de molécules d’eau de cristallisation. En revanche la courbe DSC indique un seul pic exothermique à 573 K, qui correspond à la décomposition de la partie organique du composé (I).

## Analyse de surface Hirshfeld   

**Figure 6:**
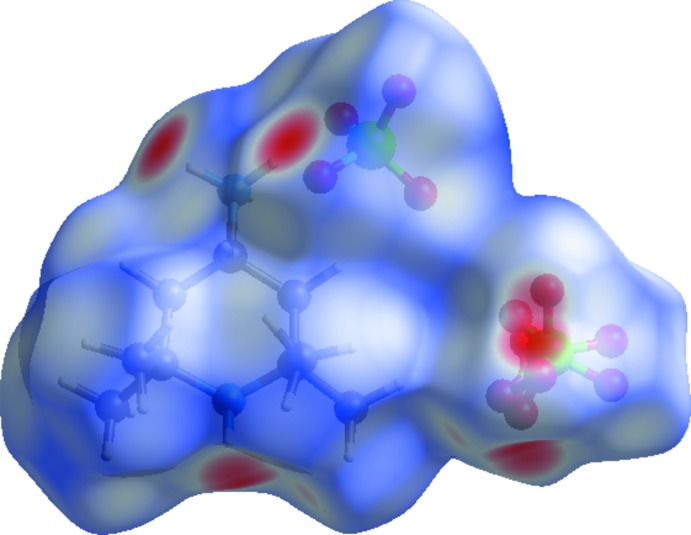
Surface Hirshfeld en mode *d*_norm_ du composé (I).

La représentation de la surface Hirshfeld du cation organique (C_9_H_22_N_2_)^2+^ et des deux anions ClO_4_^−^ de l’unité asymétrique du composé (I) permet de mettre en évidence les liaisons hydrogène et les inter­actions inter­moléculaires dans la structure cristalline (Spackman & McKinnon, 2002 ▸; Spackman & Jayatilaka, 2009 ▸). La surface Hirshfeld en modes *d*_norm_ générée à l’aide du programme *CrystalExplorer* (Wolff *et al.*, 2012 ▸) est illustrée dans la Fig. 6 ▸. Les tâches rouges correspondent aux contacts rapprochés O⋯H/H⋯O qui sont dus aux liaisons hydrogène N—H⋯O. Les zones blanches marquent les endroits où la distance séparant les atomes voisins avoisine la somme des rayons de van der Waals des atomes considérés, elles indiquent des inter­actions de type H⋯H. Les zones bleutées illustrent les domaines où les atomes voisins sont trop éloignés pour inter­agir entre eux.

**Figure 7:**
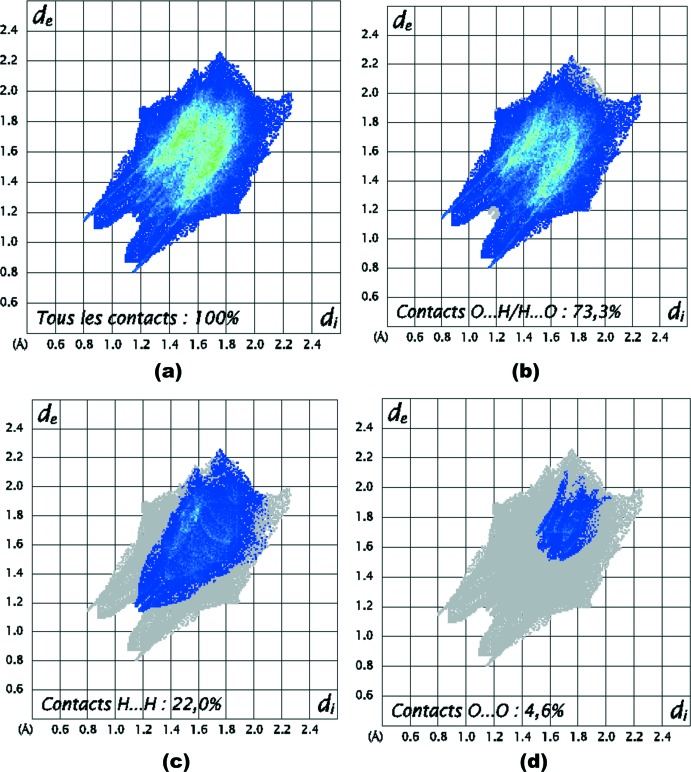
Empreintes digitales bidimensionnelles du composé (I): tous les contacts inter­moléculaires (*a*), contacts O⋯H/H⋯O (*b*), H⋯H (*c*) et O⋯O (*d*).

Les empreintes digitales 2D de la surface Hirshfeld de la structure étudiée permettent de mettre en évidence les atomes participant à des contacts rapprochés (Parkin *et al.*, 2007 ▸; Rohl *et al.*, 2008 ▸). La Fig. 7 ▸ *a* illustre l’empreinte 2D de la totalité des contacts contribuant à la surface Hirshfeld. Le graphique exposé dans la Fig. 7 ▸ *b* représente les contacts H⋯O/O⋯H entre les atomes d’hydrogène situés à l’intérieur de la surface Hirshfeld et les atomes d’oxygène situés à l’extérieur et réciproquement. Il est caractérisé par deux pointes symétriques situées en haut et à gauche et en bas à droite avec *d_e_* + *d_i_* = 2 Å. Ces données sont caractéristiques des liaisons hydrogène N—H⋯O. Elles ont la contribution la plus importante à la surface Hirshfeld totale (73,3%). Le graphique représenté dans la Fig. 7 ▸ *c* illustre l’empreinte 2D des points (*d_i_, d_e_*) associés aux atomes d’hydrogène (r_vdW_ = 1,20 Å). Il est caractérisé par une extrémité qui pointe vers l’origine selon la diagonale et qui correspond à *d_i_* ∼*d_e_* ∼ 1,2 Å, ce qui révèle la présence des contacts rapprochés H⋯H au sein du composé étudié. Ces contacts H⋯H représentent 22,0% de la totalité de tous les contacts inter­moléculaires. La décomposition de l’empreinte digitale 2D montre aussi d’autres contacts: O⋯O (4,6%, Fig. 7 ▸ *d*) et Cl⋯H/H⋯Cl (0,1%).

## Synthèse et cristallisation   

Le composé (C_9_H_22_N_2_)[ClO_4_]_2_ est obtenu en mélangeant dans l’eau, le 4-amino-2,2,6,6-tétraméthylpipéridine (pureté ≥97%, Sigma–Aldrich) et l’acide perchlorique (pureté 70%, Merck) selon les proportions molaire 1: 2. Après agitation, la solution finale est laissée évaporer à température ambiante. Après quelques jours, des cristaux incolores sous forme de parallélépipèdes commencent à apparaître. Ils ont une taille optimale pour une étude structurale.

## Affinement   

Les atomes d’hydrogène liés aux atomes de carbone ont été fixés dans leurs positions géométriques calculés en appliquant les contraintes suivantes: C—H = 0,96 Å pour le groupement –CH_3_ avec *U*_iso_(H) = 1,5*U*_eq_(C) et C—H = 0,97 Å pour le groupement –CH_2_ avec *U*_iso_(H) = 1,2*U*_eq_(C). Les données cristallographiques, les conditions de la collecte et les résultats de l’affinement de la structure du composé (I) sont regroupés dans le Tableau 2 ▸.

## Supplementary Material

Crystal structure: contains datablock(s) I. DOI: 10.1107/S2056989017012695/vm2204sup1.cif

Structure factors: contains datablock(s) I. DOI: 10.1107/S2056989017012695/vm2204Isup2.hkl

Supporting information file. DOI: 10.1107/S2056989017012695/vm2204Isup3.cml

CCDC reference: 1010810

Additional supporting information:  crystallographic information; 3D view; checkCIF report

## Figures and Tables

**Table 1:** Géométrie des liaisons hydrogène (Å, °)

*D*—H⋯*A*	*D*—H	H⋯*A*	*D*⋯*A*	*D*—H⋯*A*
N1—H1*B*⋯O5*A* ^i^	0,84 (4)	2,38 (4)	3,178 (7)	158 (3)
N1—H1*B*⋯O8*B* ^i^	0,84 (4)	2,19 (5)	2,975 (12)	155 (3)
N1—H1*A*⋯O7*B* ^ii^	0,91 (3)	2,06 (4)	2,885 (9)	150 (3)
N1—H1*A*⋯O7*A* ^ii^	0,91 (3)	2,17 (4)	3,069 (14)	169 (3)
N1—H1*A*⋯O8*B* ^ii^	0,91 (3)	2,35 (4)	3,086 (13)	138 (3)
N2—H2*A*⋯O2	0,87 (8)	2,09 (8)	2,947 (6)	168 (6)
N2—H2*B*⋯O3^iii^	0,92 (6)	2,04 (6)	2,951 (6)	171 (5)

Codes de symétrie: (i) 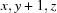; (ii) 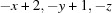; (iii) 
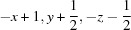
.

**Table 2:** Détails expérimentaux

Données cristallines	
Formule chimique	C_9_H_22_N_2_^2+^·2ClO_4_^−^
*M*_r_	357,18
Système cristallin, groupe d’espace	Monoclinique, *P*2_1_/*c*
Température (K)	293
*a*, *b*, *c* (Å)	11,690 (4), 8,330 (2), 16,160 (5)
β (°)	90,31 (2)
*V* (Å^3^)	1573,6 (8)
*Z*	4
Type de rayonnement	Mo *K*α
μ (mm^−1^)	0,45
Taille du cristal (mm)	0,9 × 0,54 × 0,36

Collecte de données	
Diffractomètre	Enraf–Nonius CAD-4
Correction d’absorption	ψ scan (North *et al.*,1968 ▸)
*T*_min_, *T*_max_	0,87, 0,99
Nombre de réflexions mesurées, indépendantes et observées [*I* > 2σ(*I*)]	4106, 3423, 2193
*R*_int_	0,028

Affinement	
*R*[*F* ^2^ > 2σ(*F* ^2^)], *wR*(*F* ^2^), *S*	0,061, 0,176, 1,04
Nombre de réflexions	3077
Nombre de paramètres	251
Nombre de restraints	76
Traitement des atomes d’hydrogène	H atomes traitées par un mélange d’affinement indépendant et contraint
Δρ_max_, Δρ_min_ (e Å^−3^)	0,50, −0,31

Programmes informatiques:: *CAD-4 EXPRESS* (Duisenberg, 1992 ▸; Macíček & Yordanov, 1992 ▸), *XCAD4* (Harms & Wocadlo, 1995 ▸), *SHELXS97* (Sheldrick, 2008 ▸), *SHELXL2014/7* (Sheldrick, 2015 ▸), *DIAMOND* (Brandenburg, 2006 ▸), *WinGX* (Farrugia, 2012 ▸) et *publCIF* (Westrip, 2010 ▸).

## supplementary crystallographic information

### Crystal data

**Table:**

C_9_H_22_N_2_^2+^·2ClO_4_^−^	*F*(000) = 752
*M**_r_* = 357.18	*D*_x_ = 1.508 Mg m^−^^3^
Monoclinic, *P*2_1_/*c*	Mo *K*α radiation, λ = 0.71073 Å
*a* = 11.690 (4) Å	Cell parameters from 25 reflections
*b* = 8.330 (2) Å	θ = 12–15°
*c* = 16.160 (5) Å	µ = 0.45 mm^−^^1^
β = 90.31 (2)°	*T* = 293 K
*V* = 1573.6 (8) Å^3^	Parallelepiped, colorless
*Z* = 4	0.9 × 0.54 × 0.36 mm

### Data collection

**Table:**

Enraf–Nonius CAD-4 diffractometer	2193 reflections with *I* > 2σ(*I*)
Radiation source: fine-focus sealed tube	*R*_int_ = 0.028
Graphite monochromator	θ_max_ = 27.0°, θ_min_ = 2.5°
w/2θ scans	*h* = −14→1
Absorption correction: ψ scan (North *et al.*,1968)	*k* = −1→10
*T*_min_ = 0.87, *T*_max_ = 0.99	*l* = −20→20
4106 measured reflections	2 standard reflections every 120 reflections
3423 independent reflections	intensity decay: 1%

### Refinement

**Table:**

Refinement on *F*^2^	76 restraints
Least-squares matrix: full	Hydrogen site location: mixed
*R*[*F*^2^ > 2σ(*F*^2^)] = 0.061	H atoms treated by a mixture of independent and constrained refinement
*wR*(*F*^2^) = 0.176	*w* = 1/[σ^2^(*F*_o_^2^) + (0.0865*P*)^2^ + 1.3556*P*] where *P* = (*F*_o_^2^ + 2*F*_c_^2^)/3
*S* = 1.04	(Δ/σ)_max_ < 0.001
3077 reflections	Δρ_max_ = 0.50 e Å^−^^3^
251 parameters	Δρ_min_ = −0.31 e Å^−^^3^

### Special details

**Table:**

Geometry. All esds (except the esd in the dihedral angle between two l.s. planes) are estimated using the full covariance matrix. The cell esds are taken into account individually in the estimation of esds in distances, angles and torsion angles; correlations between esds in cell parameters are only used when they are defined by crystal symmetry. An approximate (isotropic) treatment of cell esds is used for estimating esds involving l.s. planes.

### Fractional atomic coordinates and isotropic or equivalent isotropic displacement parameters (Å^2^)

**Table:**

	*x*	*y*	*z*	*U*_iso_*/*U*_eq_	Occ. (<1)
Cl1	0.50151 (7)	0.31499 (12)	−0.13350 (6)	0.0489 (3)	
Cl2	0.81520 (6)	0.14325 (11)	0.10249 (6)	0.0451 (3)	
O1	0.4382 (3)	0.1784 (4)	−0.1580 (3)	0.0826 (10)	
O2	0.4241 (2)	0.4462 (4)	−0.1214 (2)	0.0737 (9)	
O3	0.5804 (2)	0.3560 (5)	−0.1981 (2)	0.0792 (10)	
O4	0.5640 (3)	0.2872 (5)	−0.0592 (2)	0.0813 (10)	
O5A	0.7387 (5)	0.0548 (7)	0.0500 (4)	0.086 (2)	0.625 (7)
O6A	0.7578 (9)	0.2766 (13)	0.1287 (8)	0.123 (4)	0.625 (7)
O7A	0.8971 (10)	0.2623 (16)	0.1176 (14)	0.194 (6)	0.625 (7)
O8A	0.8629 (8)	0.0545 (13)	0.1667 (5)	0.115 (3)	0.625 (7)
O5B	0.777 (2)	0.071 (3)	0.1711 (9)	0.164 (8)	0.375 (7)
O6B	0.7303 (15)	0.252 (3)	0.096 (2)	0.221 (14)	0.375 (7)
O7B	0.9060 (7)	0.196 (2)	0.0523 (8)	0.090 (4)	0.375 (7)
O8B	0.8933 (9)	0.068 (2)	0.0508 (10)	0.124 (6)	0.375 (7)
N1	0.8554 (2)	0.8496 (3)	−0.09235 (18)	0.0353 (6)	
H1A	0.931 (3)	0.831 (4)	−0.100 (2)	0.028 (8)*	
H1B	0.843 (3)	0.914 (5)	−0.053 (3)	0.042 (10)*	
N2	0.4985 (3)	0.7836 (6)	−0.1322 (3)	0.0631 (10)	
H2A	0.469 (5)	0.689 (10)	−0.124 (5)	0.11 (2)*	
H2B	0.476 (4)	0.818 (7)	−0.184 (4)	0.077 (15)*	
H2C	0.480 (4)	0.831 (7)	−0.086 (2)	0.086 (19)*	
C1	0.8071 (3)	0.6875 (4)	−0.0636 (2)	0.0382 (7)	
C2	0.6774 (3)	0.7041 (4)	−0.0605 (2)	0.0430 (8)	
H2D	0.6572	0.7757	−0.0155	0.052*	
H2E	0.6441	0.5999	−0.0488	0.052*	
C3	0.6264 (2)	0.7688 (4)	−0.1410 (2)	0.0410 (7)	
H3	0.6439	0.6948	−0.1863	0.049*	
C4	0.6758 (3)	0.9335 (4)	−0.1603 (2)	0.0404 (7)	
H4A	0.6543	1.0074	−0.1166	0.048*	
H4B	0.6422	0.9723	−0.2115	0.048*	
C5	0.8058 (3)	0.9342 (4)	−0.1685 (2)	0.0365 (7)	
C6	0.8472 (3)	0.8544 (6)	−0.2482 (2)	0.0592 (10)	
H6A	0.8104	0.7521	−0.2546	0.089*	
H6B	0.9286	0.8396	−0.2453	0.089*	
H6C	0.8286	0.9215	−0.2946	0.089*	
C7	0.8506 (3)	1.1078 (5)	−0.1640 (3)	0.0579 (10)	
H7A	0.8181	1.1695	−0.2084	0.087*	
H7B	0.9324	1.1076	−0.1686	0.087*	
H7C	0.8291	1.1546	−0.1120	0.087*	
C8	0.8546 (3)	0.6613 (5)	0.0239 (3)	0.0566 (10)	
H8A	0.8321	0.7491	0.0587	0.085*	
H8B	0.9365	0.6555	0.0220	0.085*	
H8C	0.8248	0.5628	0.0459	0.085*	
C9	0.8465 (4)	0.5516 (5)	−0.1205 (3)	0.0624 (11)	
H9A	0.8348	0.4503	−0.0935	0.094*	
H9B	0.9262	0.5647	−0.1326	0.094*	
H9C	0.8030	0.5545	−0.1711	0.094*	

### Atomic displacement parameters (Å^2^)

**Table:**

	*U*^11^	*U*^22^	*U*^33^	*U*^12^	*U*^13^	*U*^23^
Cl1	0.0460 (5)	0.0553 (6)	0.0455 (5)	0.0046 (4)	−0.0021 (3)	−0.0024 (4)
Cl2	0.0418 (4)	0.0486 (5)	0.0449 (5)	0.0028 (3)	0.0007 (3)	−0.0056 (4)
O1	0.077 (2)	0.067 (2)	0.103 (3)	−0.0131 (16)	−0.0159 (18)	−0.0022 (19)
O2	0.0682 (18)	0.067 (2)	0.086 (2)	0.0199 (15)	0.0057 (15)	0.0007 (17)
O3	0.0642 (17)	0.109 (3)	0.065 (2)	−0.0054 (17)	0.0111 (14)	−0.0009 (19)
O4	0.080 (2)	0.107 (3)	0.0565 (19)	0.0245 (19)	−0.0170 (15)	−0.0021 (19)
O5A	0.104 (4)	0.066 (4)	0.086 (4)	−0.010 (3)	−0.042 (3)	−0.023 (3)
O6A	0.112 (8)	0.102 (6)	0.154 (8)	0.045 (5)	−0.056 (6)	−0.076 (6)
O7A	0.157 (9)	0.134 (9)	0.290 (18)	−0.052 (7)	0.034 (10)	−0.035 (11)
O8A	0.139 (6)	0.130 (7)	0.075 (5)	0.038 (6)	−0.045 (5)	0.024 (5)
O5B	0.237 (19)	0.18 (2)	0.072 (10)	−0.076 (18)	0.042 (13)	0.037 (10)
O6B	0.050 (8)	0.27 (3)	0.35 (4)	0.043 (12)	−0.022 (14)	0.05 (3)
O7B	0.038 (4)	0.154 (12)	0.077 (7)	−0.013 (6)	0.016 (4)	0.019 (8)
O8B	0.082 (7)	0.149 (12)	0.142 (12)	−0.017 (8)	0.040 (7)	−0.092 (10)
N1	0.0382 (14)	0.0327 (14)	0.0351 (15)	−0.0004 (10)	−0.0071 (10)	0.0018 (12)
N2	0.0418 (17)	0.081 (3)	0.067 (3)	−0.0056 (17)	−0.0101 (16)	0.013 (2)
C1	0.0445 (16)	0.0292 (15)	0.0407 (18)	−0.0008 (12)	−0.0071 (13)	0.0073 (13)
C2	0.0485 (17)	0.0421 (18)	0.0383 (19)	−0.0083 (14)	−0.0020 (13)	0.0079 (14)
C3	0.0361 (15)	0.0457 (19)	0.0410 (18)	−0.0025 (13)	−0.0067 (12)	0.0015 (15)
C4	0.0456 (17)	0.0357 (16)	0.0397 (18)	0.0034 (13)	−0.0084 (12)	0.0063 (14)
C5	0.0444 (16)	0.0342 (16)	0.0306 (16)	−0.0041 (12)	−0.0039 (11)	0.0054 (13)
C6	0.073 (2)	0.069 (3)	0.0355 (19)	0.000 (2)	0.0089 (16)	0.0007 (18)
C7	0.074 (2)	0.040 (2)	0.059 (2)	−0.0170 (18)	−0.0132 (18)	0.0145 (18)
C8	0.070 (2)	0.052 (2)	0.047 (2)	−0.0027 (17)	−0.0189 (17)	0.0178 (18)
C9	0.079 (3)	0.037 (2)	0.070 (3)	0.0173 (18)	−0.008 (2)	−0.0087 (19)

### Geometric parameters (Å, º)

**Table:**

Cl1—O1	1.413 (3)	C1—C8	1.532 (5)
Cl1—O4	1.422 (3)	C2—C3	1.526 (5)
Cl1—O2	1.433 (3)	C2—H2D	0.9700
Cl1—O3	1.438 (3)	C2—H2E	0.9700
Cl2—O5B	1.338 (11)	C3—C4	1.522 (5)
Cl2—O6B	1.349 (14)	C3—H3	0.9800
Cl2—O6A	1.366 (7)	C4—C5	1.526 (4)
Cl2—O8A	1.388 (7)	C4—H4A	0.9700
Cl2—O8B	1.390 (9)	C4—H4B	0.9700
Cl2—O7A	1.399 (9)	C5—C6	1.530 (5)
Cl2—O7B	1.411 (8)	C5—C7	1.539 (5)
Cl2—O5A	1.434 (5)	C6—H6A	0.9600
O6A—O7A	1.644 (16)	C6—H6B	0.9600
O7B—O8B	1.08 (2)	C6—H6C	0.9600
N1—C5	1.530 (4)	C7—H7A	0.9600
N1—C1	1.537 (4)	C7—H7B	0.9600
N1—H1A	0.91 (3)	C7—H7C	0.9600
N1—H1B	0.84 (4)	C8—H8A	0.9600
N2—C3	1.508 (4)	C8—H8B	0.9600
N2—H2A	0.87 (8)	C8—H8C	0.9600
N2—H2B	0.92 (6)	C9—H9A	0.9600
N2—H2C	0.870 (10)	C9—H9B	0.9600
C1—C2	1.524 (4)	C9—H9C	0.9600
C1—C9	1.531 (5)		
			
O1—Cl1—O4	111.8 (2)	C3—C2—H2E	109.0
O1—Cl1—O2	108.8 (2)	H2D—C2—H2E	107.8
O4—Cl1—O2	109.3 (2)	N2—C3—C4	108.9 (3)
O1—Cl1—O3	108.9 (2)	N2—C3—C2	109.4 (3)
O4—Cl1—O3	108.9 (2)	C4—C3—C2	110.3 (3)
O2—Cl1—O3	109.0 (2)	N2—C3—H3	109.4
O5B—Cl2—O6B	96.8 (19)	C4—C3—H3	109.4
O6A—Cl2—O8A	113.4 (7)	C2—C3—H3	109.4
O5B—Cl2—O8B	121.2 (15)	C3—C4—C5	113.6 (2)
O6B—Cl2—O8B	138.0 (18)	C3—C4—H4A	108.8
O6A—Cl2—O7A	72.9 (8)	C5—C4—H4A	108.8
O8A—Cl2—O7A	88.6 (9)	C3—C4—H4B	108.8
O5B—Cl2—O7B	150.4 (12)	C5—C4—H4B	108.8
O6B—Cl2—O7B	107.5 (16)	H4A—C4—H4B	107.7
O8B—Cl2—O7B	45.2 (8)	C4—C5—C6	113.1 (3)
O6A—Cl2—O5A	107.2 (4)	C4—C5—N1	107.5 (2)
O8A—Cl2—O5A	114.5 (5)	C6—C5—N1	110.9 (3)
O7A—Cl2—O5A	153.1 (10)	C4—C5—C7	109.7 (3)
Cl2—O6A—O7A	54.5 (6)	C6—C5—C7	109.9 (3)
Cl2—O7A—O6A	52.6 (5)	N1—C5—C7	105.5 (3)
O8B—O7B—Cl2	66.4 (7)	C5—C6—H6A	109.5
O7B—O8B—Cl2	68.4 (7)	C5—C6—H6B	109.5
C5—N1—C1	120.7 (2)	H6A—C6—H6B	109.5
C5—N1—H1A	110 (2)	C5—C6—H6C	109.5
C1—N1—H1A	104 (2)	H6A—C6—H6C	109.5
C5—N1—H1B	104 (3)	H6B—C6—H6C	109.5
C1—N1—H1B	106 (3)	C5—C7—H7A	109.5
H1A—N1—H1B	112 (3)	C5—C7—H7B	109.5
C3—N2—H2A	110 (4)	H7A—C7—H7B	109.5
C3—N2—H2B	103 (3)	C5—C7—H7C	109.5
H2A—N2—H2B	108 (6)	H7A—C7—H7C	109.5
C3—N2—H2C	112 (3)	H7B—C7—H7C	109.5
H2A—N2—H2C	100 (6)	C1—C8—H8A	109.5
H2B—N2—H2C	124 (5)	C1—C8—H8B	109.5
C2—C1—C9	112.9 (3)	H8A—C8—H8B	109.5
C2—C1—C8	109.7 (3)	C1—C8—H8C	109.5
C9—C1—C8	109.9 (3)	H8A—C8—H8C	109.5
C2—C1—N1	107.3 (2)	H8B—C8—H8C	109.5
C9—C1—N1	110.9 (3)	C1—C9—H9A	109.5
C8—C1—N1	105.9 (3)	C1—C9—H9B	109.5
C1—C2—C3	112.8 (3)	H9A—C9—H9B	109.5
C1—C2—H2D	109.0	C1—C9—H9C	109.5
C3—C2—H2D	109.0	H9A—C9—H9C	109.5
C1—C2—H2E	109.0	H9B—C9—H9C	109.5

### Hydrogen-bond geometry (Å, º)

**Table:**

*D*—H···*A*	*D*—H	H···*A*	*D*···*A*	*D*—H···*A*
N1—H1*B*···O5*A*^i^	0.84 (4)	2.38 (4)	3.178 (7)	158 (3)
N1—H1*B*···O8*B*^i^	0.84 (4)	2.19 (5)	2.975 (12)	155 (3)
N1—H1*A*···O7*B*^ii^	0.91 (3)	2.06 (4)	2.885 (9)	150 (3)
N1—H1*A*···O7*A*^ii^	0.91 (3)	2.17 (4)	3.069 (14)	169 (3)
N1—H1*A*···O8*B*^ii^	0.91 (3)	2.35 (4)	3.086 (13)	138 (3)
N2—H2*A*···O2	0.87 (8)	2.09 (8)	2.947 (6)	168 (6)
N2—H2*B*···O3^iii^	0.92 (6)	2.04 (6)	2.951 (6)	171 (5)

Symmetry codes: (i) *x*, *y*+1, *z*; (ii) −*x*+2, −*y*+1, −*z*; (iii) −*x*+1, *y*+1/2, −*z*−1/2.
